# A Narrative Review of Implant Density and Surgical Outcomes in Adolescent Idiopathic Scoliosis

**DOI:** 10.7759/cureus.114340

**Published:** 2026-08-11

**Authors:** Mutaleeb A Shobode, Anirejuoritse Bafor, Rami Rajjoub, Kirsten Tulchin-Francis, Reid C Chambers, Allen Kadado

**Affiliations:** 1 Orthopedic Surgery, Nationwide Children’s Hospital, Columbus, USA

**Keywords:** adolescent idiopathic scoliosis, cost-effectiveness, curve correction, deformity correction, functional outcome, implant density, pedicle screw density, posterior spinal fusion, scoliosis surgery, spinal instrumentation

## Abstract

Pedicle screw instrumentation is the standard of care for posterior spinal fusion in adolescent idiopathic scoliosis (AIS) because of its superior biomechanical stability and ability to facilitate 3D deformity correction, but despite widespread adoption, the optimal pedicle screw density required to achieve durable correction while minimizing complications and cost remains controversial. The purpose of this study is to synthesize available evidence evaluating the relationship between pedicle screw density and radiographic, functional, perioperative, and economic outcomes in AIS surgery. A narrative review of the literature was conducted using PubMed and Google Scholar, including English-language studies examining the association between implant density and surgical outcomes in AIS and young adult idiopathic scoliosis. Thirty studies were ultimately included, with the majority demonstrating no consistent association between higher implant density (ID) and improved radiographic correction or functional outcomes, as low-density constructs achieved comparable coronal and sagittal correction, health-related quality-of-life scores, and maintenance of correction. Conversely, higher ID was consistently associated with increased operative time, blood loss, and implant-related cost, and while select studies suggested improved correction with higher-density fixation in specific curve types or patterns, these effects diminished when curve flexibility and surgical strategy were considered. Ultimately, current evidence suggests that higher pedicle screw density does not reliably confer superior radiographic or functional outcomes in AIS, while conversely increasing operative demand and cost, meaning that low-density constructs represent a safe and effective alternative in appropriately selected patients.

## Introduction and background

The surgical management of scoliosis has evolved substantially over the past seven decades, progressing from non-instrumented fusion techniques to modern segmental spinal instrumentation dominated by pedicle screw constructs [1,2]. Early surgical approaches achieved limited deformity correction and relied heavily on prolonged external immobilization. The advent of segmental instrumentation represented a major paradigm shift, enabling controlled application of corrective forces across multiple spinal levels and improving deformity correction, fusion rates, and postoperative stability [3].

Pedicle screw instrumentation has since become the cornerstone of posterior spinal fusion for adolescent idiopathic scoliosis (AIS). The ability to achieve strong bony purchase within the vertebral pedicle provides tri-column fixation and superior resistance to pullout and torsional forces compared with hook- or wire-based systems [1,3]. These biomechanical advantages facilitate 3D deformity correction, including coronal plane realignment, restoration of sagittal alignment, and axial vertebral derotation, while minimizing implant encroachment of the spinal canal. Consequently, all-pedicle screw constructs have largely replaced hybrid instrumentation strategies in contemporary AIS surgery [4].

Despite widespread adoption of pedicle screw fixation, substantial variability persists in the number and distribution of screws used during posterior spinal fusion for AIS. Implant density (ID), commonly defined as the number of pedicle screws inserted per fused spinal level, varies considerably among surgeons and institutions [3-6]. This variability reflects differences in surgical philosophy, training, perceived biomechanical requirements, and resource availability. Although higher ID is often assumed to improve construct rigidity and corrective potential, it may also increase operative time, blood loss, implant-related complications, and overall treatment cost [7,8].

Although the terms “low-density” and “high-density” constructs are widely used, there is no universally accepted definition of ID. Nevertheless, many surgeons continue to favor higher-density constructs, particularly for larger or stiffer curves, based on the belief that greater fixation provides superior deformity correction and long-term stability despite conflicting evidence [3,7-12]. Studies have classified ID using different thresholds based on screws per fused level, anchor density, or screw distribution strategies, making direct comparison across studies challenging [3,13-18].

The assumption that greater ID translates into superior clinical outcomes has increasingly been questioned. Several studies have demonstrated comparable radiographic correction, maintenance of correction, and functional outcomes using lower-density constructs, suggesting that maximal screw density may not be necessary for effective deformity correction in all patients [7-10]. Emerging evidence further suggests that curve-specific factors, including curve magnitude, flexibility, Lenke classification, sagittal alignment, and correction strategy, may exert a greater influence on surgical outcomes than ID alone. Interpretation of the literature is further complicated by heterogeneity in ID definitions, patient populations, surgical techniques, and reported outcome measures [13-18].

Beyond deformity correction, ID has important implications for patient safety, healthcare resource utilization, and value-based spine care. Identifying instrumentation strategies that achieve durable correction while minimizing operative morbidity and implant-related costs has therefore become an increasingly important clinical objective.

Accordingly, this narrative review examines the relationship between pedicle screw ID and surgical outcomes in AIS, with particular focus on radiographic correction, functional outcomes, perioperative parameters, complication profiles, and cost-effectiveness.

## Review

Methods

A narrative literature review was conducted to evaluate the relationship between pedicle screw ID and surgical outcomes in AIS. Electronic literature searches were performed in PubMed and Google Scholar between January 1, 2000, and March 31, 2025, with the final search completed on March 31, 2025.

The search strategy combined Medical Subject Headings (MeSH), where applicable, and free-text keywords using Boolean operators. The primary search terms included “adolescent idiopathic scoliosis”, “AIS”, “pedicle screw density”, “implant density”, “anchor density”, “pedicle screw construct”, “low-density”, “high-density”, and “posterior spinal fusion”. These terms were combined using the Boolean operators AND and OR. Reference lists of all eligible articles and relevant review papers were also manually screened to identify additional studies that may not have been captured during the electronic search.

Studies published in English that evaluated the association between ID and radiographic, functional, perioperative, biomechanical, or economic outcomes following posterior spinal fusion for AIS were considered eligible. Both AIS and young adult idiopathic scoliosis populations were included because of the overlap in surgical principles and instrumentation strategies between these groups [3,11,12]. Eligible study designs included systematic reviews and meta-analyses, randomized controlled trials, prospective and retrospective comparative studies, cohort studies, biomechanical investigations, and cost-effectiveness analyses. Case reports, conference abstracts, purely technical descriptions without clinical outcome data, editorials, expert opinions, and non-English language publications were excluded.

Titles and abstracts identified through the search strategy were screened independently by two reviewers. Full-text articles considered potentially eligible were subsequently assessed independently by the same reviewers for inclusion. Any disagreements regarding study eligibility were resolved through discussion and consensus. For each eligible study, data were extracted on study design, sample size, patient population, curve characteristics, ID definition, comparator groups, radiographic, functional, perioperative, biomechanical, safety, and economic outcomes, together with the principal findings relevant to the objectives of this review. Data extraction was performed independently by the two reviewers, with discrepancies resolved through discussion and consensus. Where outcome measures or study characteristics were incompletely or inconsistently reported, the available data were summarized qualitatively, and no attempts were made to impute missing information.

**Figure 1:**
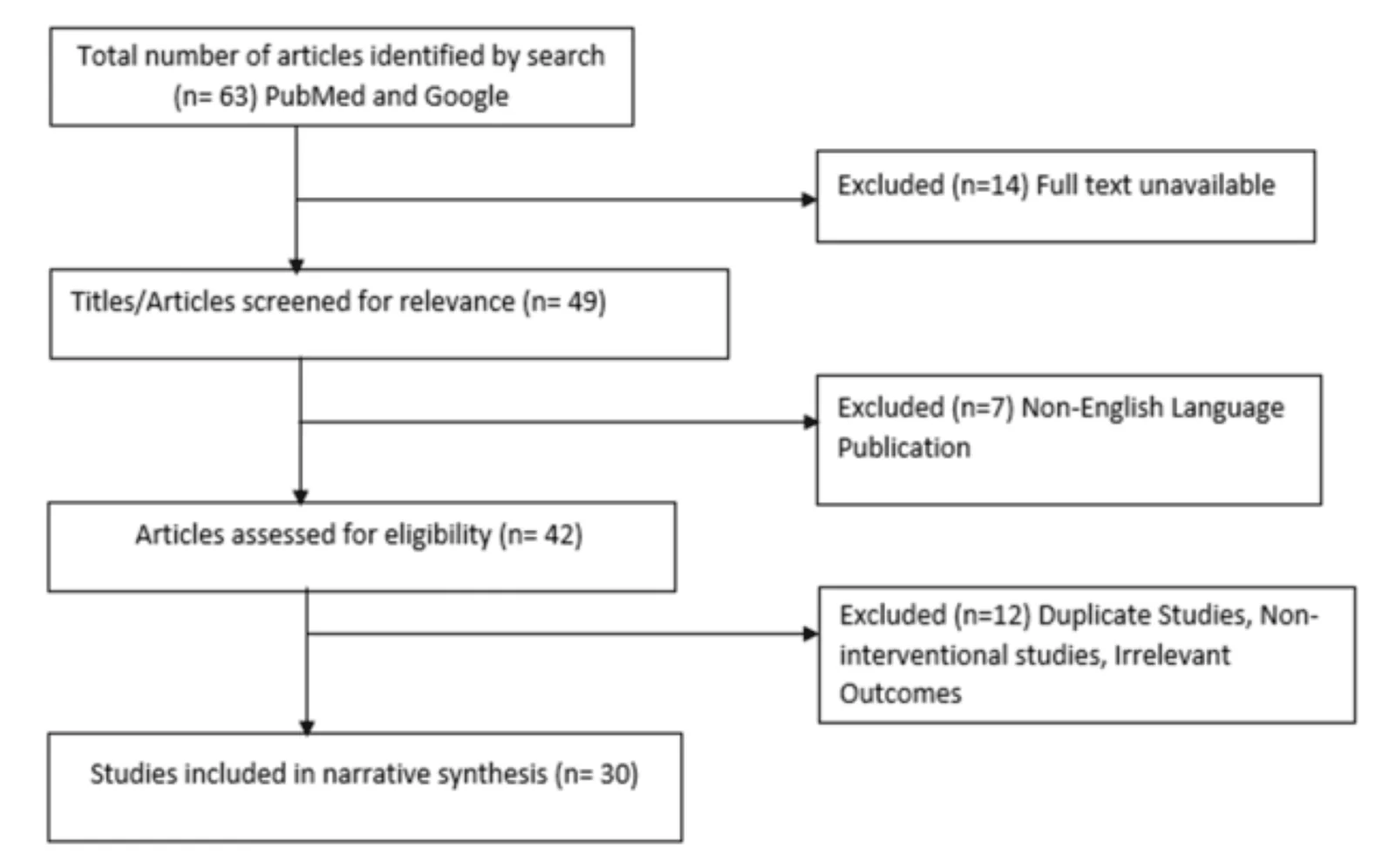
Literature identification and study selection flow diagram. Flow diagram illustrating the identification, screening, eligibility assessment, and inclusion of studies evaluating pedicle screw ID in AIS surgery. Sixty-three potentially relevant studies were identified, with 30 studies ultimately included in the narrative review. AIS, adolescent idiopathic scoliosis; ID, implant density

Because this was a narrative review, studies were selected to provide a comprehensive overview of the available evidence rather than to facilitate formal quantitative synthesis. Consequently, no formal assessment of methodological quality or risk of bias was undertaken. Given the considerable heterogeneity in ID definitions, patient populations, curve characteristics, surgical techniques, and reported outcome measures, a narrative synthesis was considered the most appropriate approach for summarizing and interpreting the current literature [3,10,13]. The study identification and selection process is summarized in Figure 1.

Results

The included literature comprised a heterogeneous body of evidence evaluating the relationship between pedicle screw ID and surgical outcomes in AIS surgery. The studies included systematic reviews, randomized controlled trials, retrospective and prospective cohort studies, biomechanical modeling investigations, and economic analyses. These studies examined multiple outcome domains, including radiographic deformity correction, functional and health-related quality of life outcomes, operative duration, intraoperative blood loss, complication profiles, and cost-effectiveness.

**Figure 2:**
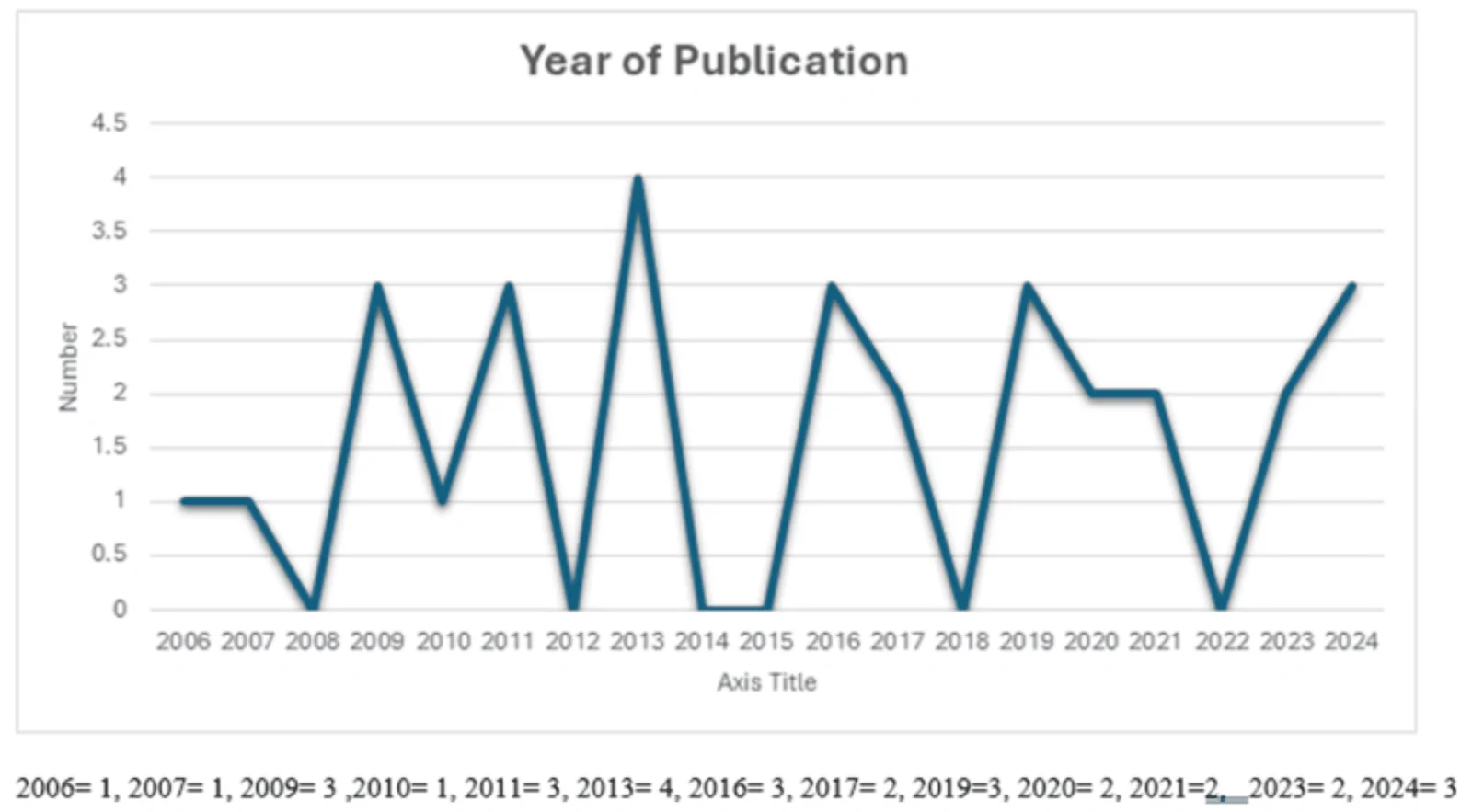
Temporal distribution of included studies by year of publication. Line graph demonstrating the annual distribution of included studies evaluating ID in AIS surgery between 2006 and 2024. Increased publication activity was observed after 2010, reflecting growing interest in optimization of pedicle screw construct density and value-based deformity correction strategies. AIS, adolescent idiopathic scoliosis; ID, implant density

**Figure 3:**
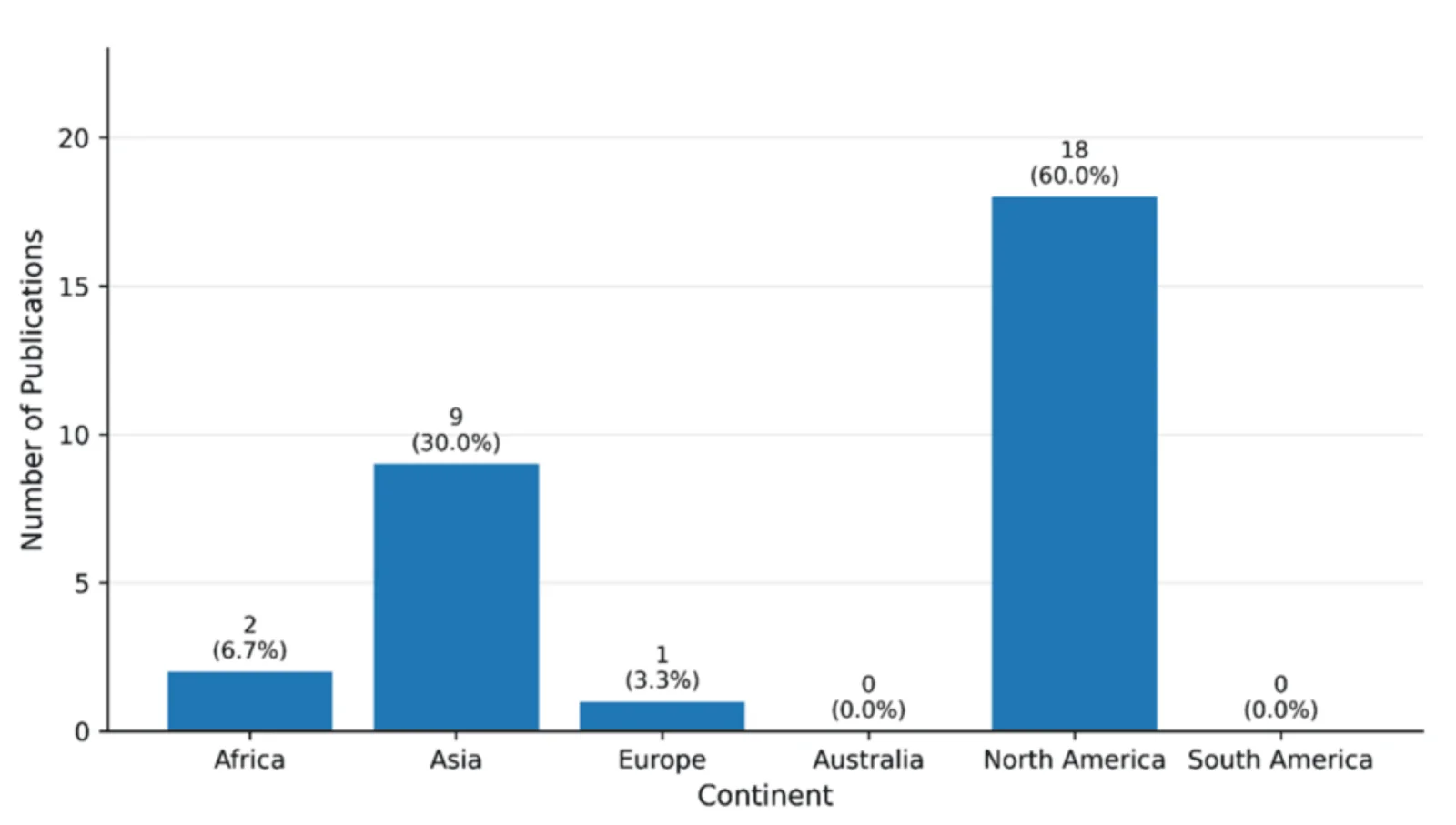
Geographic distribution of included studies. Bar chart illustrating the regional origin of studies evaluating pedicle screw ID in AIS surgery. Most publications originated from North America and Asia, with fewer studies from Europe and Africa, and no eligible studies from Australia or South America. This distribution highlights regional variation in research output and may reflect differences in surgical practice patterns, resource availability, and research infrastructure. AIS, adolescent idiopathic scoliosis; ID, implant density

**Figure 4:**
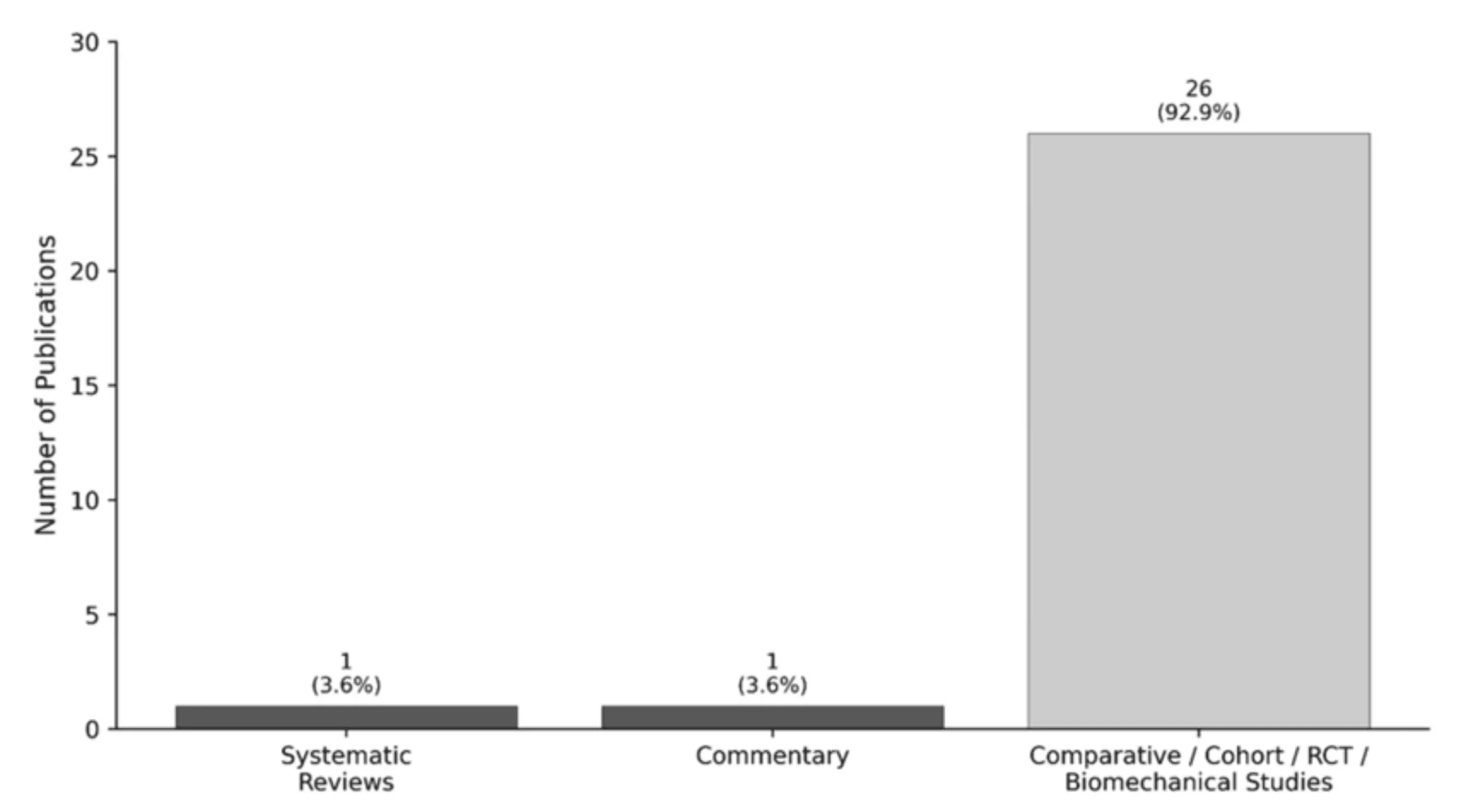
Distribution of included studies by study design. Bar chart illustrating the methodological composition of included studies evaluating pedicle screw ID in AIS surgery. Comparative cohort studies and retrospective analyses represented the majority of included publications, with fewer randomized controlled trials, biomechanical studies, systematic reviews, and commentaries. AIS, adolescent idiopathic scoliosis; ID, implant density; RCT, randomized controlled trial

Substantial variability existed across studies with respect to ID definitions, curve characteristics, surgical techniques, correction strategies, and reported outcome measures. Despite this heterogeneity, several consistent themes emerged. Most studies reported no clear independent association between higher ID and improved radiographic or functional outcomes. In contrast, denser constructs were more consistently associated with increased operative time, greater blood loss, and higher implant-related costs. The characteristics and principal findings of the included studies are summarized in Table 1, while the synthesized effects of ID across major clinical and perioperative outcome domains are presented in Table 2. Variability in ID definitions across studies is summarized in Table 3. The literature selection process is illustrated in Figure 1, and the temporal, geographic, and methodological distribution of included studies are presented in Figures 2-4.

**Table 1:** Summary of key studies evaluating ID and outcomes in AIS. Summary of representative studies examining the relationship between pedicle screw ID and radiographic, functional, perioperative, biomechanical, and economic outcomes in AIS surgery. AIS, adolescent idiopathic scoliosis; ID, implant density; LIV, lowest instrumented vertebra; SRS, Scoliosis Research Society

Author	Study design	Sample size	Curve type	ID definition	Key outcomes assessed	Main findings
Tannous et al. (2018) [7]	Retrospective cohort	35	Mixed AIS	Mean 1.2 screws per level	LIV angle kyphosis curve correction cost	Low-density constructs achieved similar correction with significant cost savings
Sultan et al. (2024) [8]	Prospective cohort	60	Mixed Lenke	Mean 1.2 ± 0.23 screws per level	Correction rate, blood loss, operative time	Low-density constructs achieved correction comparable to high-density series with reduced operative burden
Wang et al. (2012) [9]	Biomechanical simulation	8 cases	Thoracic AIS	Low vs high density at identical fusion levels	Cobb correction axial rotation implant forces	Equivalent correction achieved with low-density constructs and lower implant vertebra forces
Lertudomphonwanit et al. (2022) [13]	Retrospective comparative study	122	Lenke 1 and 2 AIS	Lower- versus higher-density constructs	3D deformity correction	ID did not significantly influence 3D deformity correction in Lenke 1 and 2 AIS curves treated without Ponte osteotomies
Skalak et al. (2021) [14]	Retrospective cohort	30	Lenke 2	Mean 1.6 anchors per level	Radiographic correction	High ID was not predictive of postoperative curve correction
Shen et al. (2017) [15]	Comparative cohort	62	Lenke 1	Low <1.61 vs high >1.61 screws per level	Cobb angle, thoracic kyphosis, SRS 22	Low-density and high-density constructs achieved comparable correction and functional outcomes
Yeh et al. (2019) [16]	Retrospective cohort	74	AIS	Mean 1.6 screws per level	Cobb angle correction	Comparable correction achieved without high-density instrumentation
Kilinc et al. (2019) [17]	Retrospective cohort	52	Lenke 1B and 1C	Mean 1.3 screws per level	Radiographic correction	Low-density constructs achieved correction similar to higher-density reports
Mostafa et al. (2024) [18]	Randomized controlled trial	20	Mixed AIS	Low ≤1.5 vs high >1.5 screws per level	Cobb correction, quality of life, operative time	Low-density constructs provided equivalent correction with shorter operative time and hospital stay
Chotigavanichaya et al. (2023) [19]	Retrospective comparative study with inverse probability weighting	120	Small and flexible AIS curves	Different pedicle screw density pattern	Radiographic correction, operative burden	Lower-density constructs achieved comparable deformity correction with reduced implant utilization and operative burden
Aoun et al. (2024) [20]	Systematic review and meta-analysis	18	Mixed AIS	Low-density versus high-density constructs	Radiographic correction, clinical outcomes, perioperative parameters	Meta-analysis demonstrated no consistent superiority of high-density constructs for radiographic or functional outcomes, while higher density increased operative burden and cost

**Table 2:** Effect of ID across clinical outcome domains in AIS surgery. Synthesis of the reported effects of low-density and high-density pedicle screw constructs across major radiographic, functional, perioperative, safety, and economic outcome domains in AIS surgery. Interpretations are based on the synthesis of included studies and reflect overall trends rather than the results of individual trials. Outcome domains represent commonly reported clinical and perioperative endpoints in AIS surgery. AIS, adolescent idiopathic scoliosis; ID, implant density; SRS, Scoliosis Research Society

Outcome domain	Effect of high-density constructs	Effect of low-density constructs	Overall interpretation
Radiographic correction [7-9,13-20]	No consistent superiority across studies	Comparable correction in most series	ID alone is not a dominant predictor
Implant-related cost [7,20]	Higher total implant cost	Substantial cost savings	Lower density is more cost-effective
Operative duration [8,18-20]	Longer operative time	Shorter operative time	Lower density improves operative efficiency
Intraoperative blood loss [8,19,20]	Increased blood loss	Reduced blood loss	Lower density reduces perioperative demand
Functional outcomes [15,18,19]	Similar SRS scores	Similar SRS scores	Patient-reported outcomes equivalent
Loss of correction [15,18,21]	Similar rates	Similar rates	Stability maintained with lower density
Overall safety profile [20]	Comparable major complication rates	Comparable major complication rates	Reduced screw number does not compromise safety

**Table 3:** Definitions of ID across included studies. Comparison of definitions and classification methods used to describe low-density and high-density pedicle screw constructs across included studies. Considerable heterogeneity in ID definitions was observed across the literature. Definitions of ID varied substantially across studies and included screws per fused level, anchor density, and screw distribution strategies, limiting direct comparison across the literature. AIS, adolescent idiopathic scoliosis; ID, implant density

Study	Curve type	Low-density definition	High-density definition
Bharucha et al. (2013) [3]	Thoracic AIS	Selective screw strategy	Segmental screw strategy
Tannous et al. (2018) [7]	Mixed AIS	Mean 1.2 screws/level	Maximal density construct
Sultan et al. (2024) [8]	Mixed Lenke AIS	Mean 1.2 ± 0.23 screws/level	Historical high-density constructs
Lertudomphonwanit et al. (2022) [13]	Lenke 1/2 AIS	Lower-density pattern	Higher-density pattern
Skalak et al. (2021) [14]	Lenke 2 AIS	Lower anchor density	Higher anchor density
Shen et al. (2017) [15]	Lenke 1 AIS	<1.61 screws/level	>1.61 screws/level
Yeh et al. (2019) [16]	AIS	Mean 1.6 screws/level	Higher-density constructs
Kilinc et al. (2019) [17]	Lenke 1B/1C AIS	Mean 1.3 screws/level	Published high-density constructs
Mostafa et al. (2024) [18]	Mixed AIS	≤1.5 screws/level	>1.5 screws/level
Larson et al. (2016) [21]	Mixed AIS	1.48 screws/level	1.8 screws/level
Chen et al. (2013) [22]	Lenke 5 AIS	Lower-density constructs	Higher-density constructs
Li et al. (2009) [23]	Lenke 1 AIS	Interval screw placement	Consecutive screw placement
Quan and Gibson (2010) [24]	Thoracic AIS	Limited screw density	Consecutive screw placement

Study Characteristics

The literature identification and selection process is illustrated in Figure 1. The included studies comprised systematic reviews, randomized controlled trials, retrospective and prospective cohort studies, biomechanical investigations, and economic analyses evaluating pedicle screw ID in AIS surgery. Detailed study characteristics and principal findings are summarized in Table 1. The overall influence of ID across major outcome domains is synthesized in Table 2, while differences in ID definitions used across studies are summarized in Table 3. The temporal distribution of publications, geographic origin of studies, and distribution of study designs are presented in Figures 2-4, respectively.

ID and Functional Outcome

The influence of pedicle screw ID on clinical and functional outcomes in AIS remains controversial, with heterogeneous findings reported across the literature [22]. Although higher-density instrumentation is theoretically expected to provide greater corrective capability and overall fixation rigidity, most contemporary studies have shown that low-density pedicle screw configurations achieve radiographic and functional outcomes comparable to those obtained with higher-density instrumentation.

Several clinical studies support the effectiveness of lower-density instrumentation strategies. Sultan et al. [8] reported a mean screw density of 1.2 ± 0.23 screws per fused level, achieving a mean correction angle of 43.9° ± 15.39°, a correction rate of 71.6% ± 9.12%, and minimal loss of correction at follow-up. Similarly, Shen et al. [15] evaluated 62 Lenke 1 AIS patients treated with posterior spinal fusion using either low-density or high-density screw configurations. Both groups demonstrated comparable correction of the major thoracic curve and thoracic kyphosis, with no significant differences in transfusion requirements, hospital stay, or Scoliosis Research Society (SRS)-22 scores, suggesting that reduced ID does not compromise deformity correction or patient-reported outcomes. Comparable findings were reported by Tannous et al. [7], who described equivalent radiographic correction and SRS-30 outcomes using a mean screw density of 1.2 screws per level.

Additional cohort studies have reported similar observations. Yeh et al. [16] found no meaningful association between increasing anchor density and improved postoperative correction despite a mean screw density of 1.6 screws per level. Kilinc et al. [17] likewise reported satisfactory correction using a mean density of 1.3 screws per level in Lenke 1B and 1C curves, reinforcing the effectiveness of limited screw strategies in selected deformities. More recently, Lertudomphonwanit et al. [13] noted that ID did not significantly influence 3D deformity correction in Lenke 1 and 2 AIS curves treated without Ponte osteotomies, further supporting the concept that maximal instrumentation may not be routinely necessary.

Prospective and randomized studies have also reinforced the equivalence of functional outcomes between ID strategies. Mostafa et al. [18] conducted a randomized clinical trial comparing low-density and high-density instrumentation strategies in AIS patients undergoing posterior spinal fusion. Both groups achieved comparable postoperative curve correction and quality of life outcomes, while the low-density cohort demonstrated significantly shorter operative times, fewer implanted screws, reduced hospital stay, and greater resource efficiency. Similarly, another study reported that lower-density pedicle screw patterns in flexible AIS deformities achieved radiographic outcomes comparable to those of higher-density fixation while reducing the overall cost of surgery [24].

Biomechanical studies further support these clinical findings. Wang et al. [9] used computational modeling to compare low-density and high-density pedicle screw configurations at identical fusion levels. Despite the use of more screws and greater bilateral fixation in the high-density group, no significant differences were observed in Cobb angle correction or apical axial vertebral rotation. Notably, high-density instrumentation generated substantially greater implant vertebra forces while intervertebral forces remained similar, suggesting no clear biomechanical advantage to increasing screw density. La Barbera et al. [10] similarly found that correction objectives and screw distribution patterns exerted greater influence on deformity correction than absolute ID alone.

In contrast, some studies have reported modest benefits with higher-density instrumentation in selected curve patterns. Mostafa et al. [18] observed greater correction in Lenke 1 and 2 curves treated with high-density instrumentation, although this relationship was not evident in Lenke 5 curves. These findings suggest that ID may exert greater influence in larger or stiffer thoracic deformities while offering limited benefit in more flexible curves. Chen et al. [22] also demonstrated a correlation between ID and correction of thoracolumbar and lumbar curves; however, this relationship weakened after adjustment for curve flexibility using the method described by Vora et al. [25].

Overall, available data indicate that the relationship between ID and functional outcome in AIS is complex and highly context-dependent. Curve magnitude, flexibility, Lenke classification, sagittal alignment, correction objectives, implant distribution method, and surgical techniques appear to exert greater influence on postoperative outcomes than ID alone. Consequently, routine use of uniformly high-density instrumentation does not appear justified for all AIS patients. Instead, ID should be individualized according to curve characteristics, correction goals, and patient-specific anatomy to optimize both clinical outcome and operative efficiency.

ID and Cost-Effectiveness

There is broad consensus in the literature that increasing pedicle screw ID is associated with substantially higher surgical costs in AIS surgery [7,20]. Because instrumentation represents a major contributor to the overall cost of posterior spinal fusion, the number and distribution of pedicle screws have important economic implications for both healthcare systems and individual patients. The direct relationship between implant utilization and procedural cost has been consistently reported across clinical series, comparative studies, and large database analyses [7,20].

Several studies have quantified the financial burden associated with higher-density instrumentation strategies. Skalak et al. [14] evaluated 30 AIS patients and reported a mean anchor density of 1.6 ± 0.2 anchors per level with an average ID of 80 ± 10%. Pedicle screws accounted for 92 ± 8% of all anchors used, and the mean implant cost alone was $14,463 ± 2,683, underscoring the substantial contribution of instrumentation to total surgical expenditure. Similarly, Shen et al. [15] compared low-density and high-density pedicle screw configurations in Lenke 1 AIS and found that the high-density cohort required substantially more screws than the low-density cohort (19.6 vs 15.1 screws), resulting in markedly increased implant costs of $13,577 compared with $10,191, respectively.

The economic impact of ID has been further highlighted in large-scale health system analyses. Larson et al. [21] analyzed 5,710 inpatient AIS procedures and compared standard density instrumentation of 1.48 screws per level with high-density instrumentation of 1.8 screws per level. The authors estimated that reducing ID by approximately 3.2 screws per patient could prevent nearly 932 misplaced screws and between 21 and 88 revision procedures annually in the United States. This reduction translated into projected annual cost savings of approximately $11-$20 million, representing 4-7% of total AIS hospitalization expenditures. These findings emphasize the broader economic implications of implant selection beyond the direct cost of instrumentation alone.

Patient-level analyses similarly support the financial advantages of lower-density instrumentation strategies. Tannous et al. [7] reported that low-density screw configurations achieved radiographic and functional outcomes comparable to high-density instrumentation while reducing implant costs by nearly $9,000 per patient. The total implant expenditure in the low-density group was $520,500 compared with an estimated $834,500 for the corresponding high-density instrumentation, resulting in substantial cumulative savings without compromise in deformity correction or patient-reported outcomes.

More recent investigations continue to support selective screw fixation to reduce cost in AIS surgery. They noted that lower-density screw patterns in flexible AIS deformities reduced implant utilization and perioperative demands while maintaining comparable radiographic correction. Similarly, Lertudomphonwanit et al. [13] reported no clear radiographic advantage to increased ID in Lenke 1 and 2 curves, suggesting that routine maximal instrumentation may expose patients and healthcare systems to additional cost without proportional clinical benefit.

The economic implications of ID are increasingly relevant in the era of healthcare resource utilization, where optimizing clinical outcomes while minimizing unnecessary expenditure has become a central objective of modern spine surgery. Although higher ID predictably increases implant-related costs, duration of surgery, and resource utilization, the lowest effective density required to maintain safe and durable correction remains incompletely defined. Existing studies nevertheless suggest that selective use of lower-density instrumentation strategies may provide substantial economic benefit while ensuring acceptable radiographic correction and functional outcome. These findings support a more individualized and evidence-informed approach to ID selection in AIS surgery. The consistent association between higher ID and increased implant-related cost across included studies is summarized in Table 2.

ID and Surgery Duration and Blood Loss

Higher pedicle screw ID has consistently been associated with longer duration of surgery and greater intraoperative blood loss in AIS surgery. The placement of additional pedicle screws increases the time required for pedicle preparation, screw insertion, fluoroscopic or navigational verification, rod assembly, and final instrumentation optimization. More extensive instrumentation may also require greater surgical exposure and soft tissue dissection, contributing to operative efficiency.

Several clinical studies have described this relationship. Sultan et al. [8] reported a significant positive correlation between ID, operative duration, and intraoperative blood loss in a prospective cohort of 60 AIS patients aged 10-16 years who underwent posterior spinal fusion for curves ranging from 45° to 90° across multiple Lenke classifications. Despite the increased operative complexity associated with higher-density instrumentation, the authors observed no corresponding improvement in correction rate or maintenance of correction, suggesting limited additional clinical benefit from maximal instrumentation. Comparable findings have been reported in multiple retrospective and comparative series. Tannous et al. [7] noted that low-density screw configurations achieved similar radiographic and functional outcomes while reducing implant utilization and operative resource consumption. Mostafa et al. [18] similarly reported significantly shorter operative times and reduced hospital stay in the low-density cohort compared with the high-density group, despite equivalent postoperative deformity correction and quality of life outcomes.

Studies on biomechanical and deformity correction procedures further support these observations. La Barbera et al. [10] found that correction objectives and screw distribution patterns exert greater influence on deformity correction than absolute screw density, suggesting that increasing implant number alone may unnecessarily increase operative complexity without proportionate gains in correction. Lertudomphonwanit et al. [13] also found no significant improvement in 3D correction with higher ID in Lenke 1 and 2 AIS curves treated without Ponte osteotomies, reinforcing concerns regarding the efficiency of maximal instrumentation strategies.

Increased ID may also influence perioperative morbidity indirectly through prolonged anesthesia exposure and greater intraoperative blood loss. Although major complication rates appear comparable between low-density and high-density instrumentation strategies, minimizing surgery duration and blood loss is clinically important, particularly in younger patients and in complex deformity surgery where cumulative surgical stress may be substantial.

Overall, available evidence indicates that lower-density pedicle screw instrumentation strategies are consistently associated with shorter surgical duration, blood loss, and improved operative efficiency with acceptable deformity correction and short-term clinical outcomes. These findings support the selective use of lower-density instrumentation strategies as part of an individualized and value-conscious approach to AIS surgery. Comparative perioperative findings related to operative duration and intraoperative blood loss are summarized in Tables 1, 2.

ID and Complications

Most clinical studies report no significant difference in overall major complication rates, reoperation rates, or loss of correction when comparing low-density and high-density pedicle screw strategies in AIS. Across multiple series, rates of infection, implant failure, and radiographic deterioration appear broadly comparable between fixation approaches, suggesting that reduced ID does not compromise stability or durability in appropriately selected patients.

Nevertheless, increasing ID increases cumulative exposure to screw-related risks. Each additional pedicle screw insertion carries a potential risk of malposition, pedicle breach, vascular injury, or neurological injury, particularly in the thoracic spine, where pedicles are smaller and more anatomically variable. Although catastrophic neurological complications are uncommon, the cumulative probability of screw-related adverse events increases as the total number of screws placed increases.

Several recent studies support a more selective approach to implant placement. Lertudomphonwanit et al. [13] found no clear advantage of higher ID for 3D correction in Lenke 1 and 2 curves, suggesting that additional screws may increase exposure without proportional clinical benefit in selected patients. Mostafa et al. [18] similarly proved comparable outcomes between low-density and high-density screw strategies, without evidence that reduced screw number increased loss of correction or major complications. Others reported that lower-density screw patterns in flexible AIS deformities maintained satisfactory radiographic correction with better perioperative efficiency.

By reducing the number of pedicle screws, low-density fixation strategies may mitigate exposure to screw-related morbidity while preserving effective deformity correction. This risk reduction method is especially relevant in anatomically challenging regions, smaller thoracic pedicles, and flexible curves where maximal fixation may not be required. Importantly, available evidence suggests that careful reduction in screw number does not translate into higher rates of loss of correction, implant failure, or early clinical deterioration when patients are appropriately selected.

Overall, current data indicate that while overall complication rates appear similar between low-density and high-density screw strategies, judicious reduction in ID has the potential to reduce screw-related morbidity without sacrificing surgical effectiveness. These findings support a selective and individualized approach to ID rather than routine use of maximal screw placement. The overall effect of ID on complication profiles and fixation durability is summarized in Table 2.

Discussion

This narrative review shows that most of the contemporary evidence does not support a routine advantage of high pedicle screw ID in the surgical management of AIS. Across multiple outcome domains, including radiographic correction, functional outcomes, operative duration, blood loss, complication profiles, and cost-effectiveness, low-density pedicle screw strategies consistently achieve outcomes comparable to those of higher-density fixation strategies in appropriately selected patients [7-10,13-17,23]. These findings challenge the longstanding assumption that increasing screw number alone necessarily translates into superior deformity correction or improved clinical outcome.

The findings summarized in this review should be interpreted in the context of the level of evidence supporting each outcome. While randomized controlled trials and recent systematic reviews provide higher-level evidence for selected radiographic and functional outcomes, much of the available literature consists of retrospective observational cohort studies, biomechanical investigations, and economic analyses. Consequently, conclusions regarding ID should be interpreted alongside the strengths and limitations of each study design rather than as uniformly weighted evidence.

An important observation across the literature is the marked heterogeneity in ID definitions, patient populations, curve characteristics, and surgical strategies. This variability complicates direct comparison between studies and likely contributes to many of the conflicting conclusions reported in earlier literature. Importantly, when studies account for curve-specific factors such as preoperative magnitude, flexibility, Lenke classification, sagittal alignment, and correction objectives, the independent influence of ID on outcome appears substantially reduced [10,13,20]. Several contemporary investigations suggest that curve behavior and correction procedure exert a greater influence on postoperative alignment than absolute screw number alone [10,13].

The collective radiographic and functional evidence suggests that satisfactory 3D deformity correction can frequently be achieved without maximal screw placement. Multiple cohort studies and randomized investigations have described comparable correction rates, maintenance of correction, and SRS-based functional outcomes using lower-density screw configurations [7,8,15-17,23]. More recent studies further support this concept, demonstrating that selective screw strategies in appropriately chosen AIS curves can maintain effective correction while reducing implant utilization and operative cost [13,24]. Biomechanical investigations similarly demonstrate that increasing ID does not necessarily improve correction mechanics. Wang et al. [9] showed that high-density screw configurations generated greater implant vertebra forces without producing superior correction, while La Barbera et al. [10] demonstrated that screw distribution patterns and correction objectives may be more influential than density itself. These findings support the growing concept that strategic screw placement may be more important than routine maximal fixation.

From a perioperative and healthcare systems perspective, ID has important practical implications. Higher-density screw configurations are consistently associated with longer time of surgery, increased blood loss, greater implant utilization, and substantially higher surgical cost [7,8,18,23]. In contrast, lower-density screw strategies appear capable of improving operative efficiency and reducing healthcare expenditure [26,27]. These observations are increasingly relevant in the modern era of cost-conscious spine care, where balancing clinical effectiveness with resource utilization has become central to surgical decision-making.

At the same time, the available evidence suggests that ID should not be approached in an overly simplistic manner. Some studies have reported modest radiographic advantages with higher-density fixation strategies in selected situations, particularly in larger, stiffer thoracic deformities and certain Lenke 1 and 2 patterns [18,19,21]. These benefits, however, appear limited and highly context-dependent. The current literature therefore does not support routine use of uniformly high-density screw configurations across all AIS patients [28]. Rather, ID should be individualized according to deformity stiffness, curve morphology, and surgical objectives.

Another important consideration is complication avoidance. Although major complication rates appear broadly comparable between low-density and high-density screw configurations, increasing screw number inevitably increases cumulative exposure to screw-related risks, including malposition, pedicle breach, and neurological injury [20]. Reducing unnecessary screw placement may therefore represent a practical step for minimizing implant-related morbidity while preserving effective correction, particularly in anatomically challenging thoracic pedicles.

Available contemporary literature supports a gradual shift away from uniformly high-density screw placement toward a more selective and evidence-informed approach to pedicle screw utilization in AIS surgery. Current evidence suggests that screw distribution strategy and curve behavior may exert greater influence on deformity correction and clinical outcome than absolute screw number alone. Optimizing ID according to curve behavior and correction techniques may allow surgeons to maintain good outcomes while reducing operative demand, implant-related risk, and cost [26-30].

**Figure 5:**
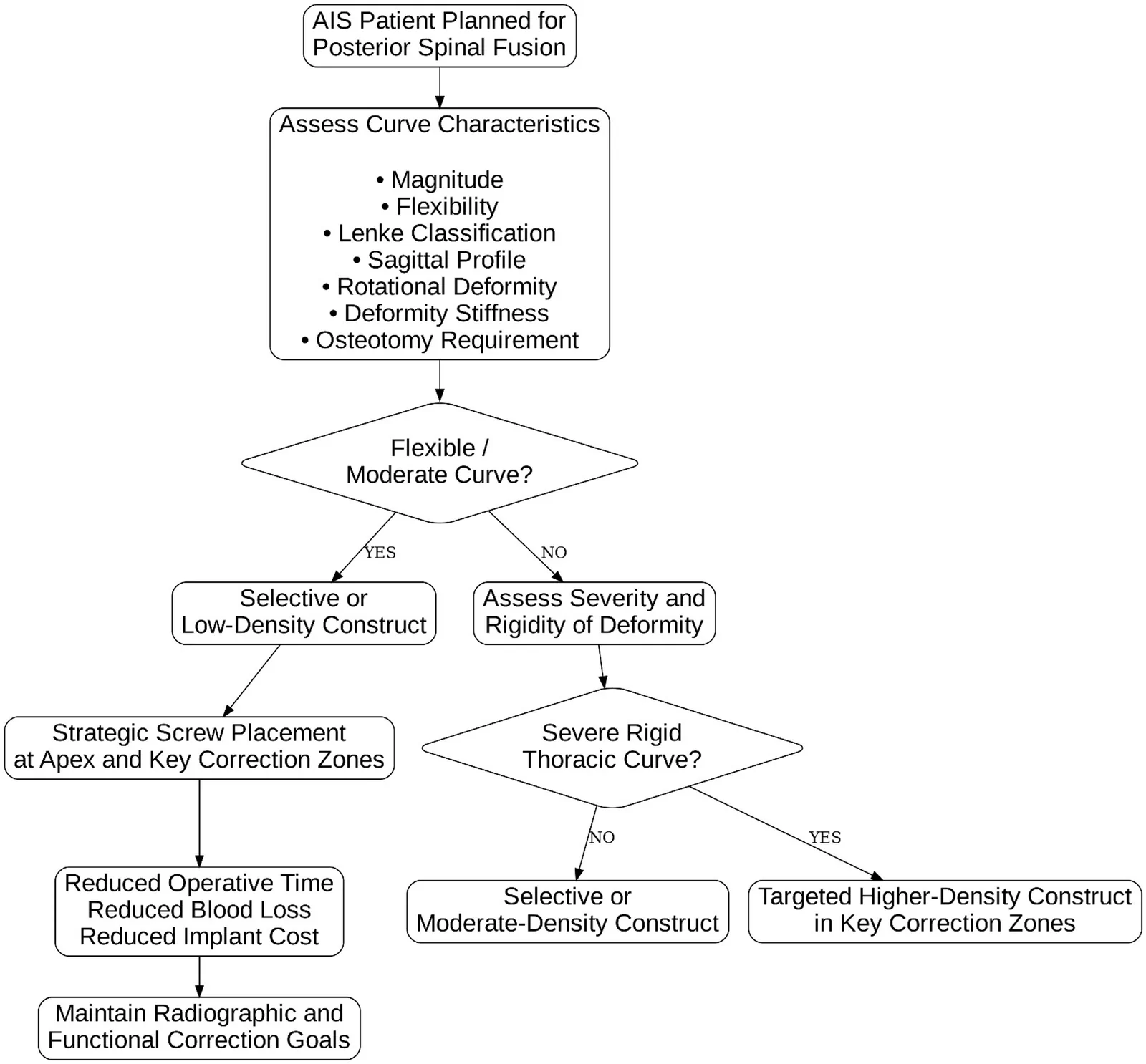
Proposed clinical decision algorithm for ID selection in AIS surgery. ID selection should be individualized according to curve magnitude, flexibility, Lenke classification, sagittal alignment, and deformity stiffness. Flexible and moderate curves may be effectively managed with selective low-density instrumentation, while higher-density constructs may be reserved for severe rigid deformities requiring greater corrective control. AIS, adolescent idiopathic scoliosis; ID, Implant density

Future prospective investigations using standardized definitions of ID, stratification by Lenke classification and curve flexibility, and incorporation of long-term patient-reported outcomes will be essential to establish more refined and evidence-based guidelines for implant utilization in AIS surgery [10,11,13]. A proposed clinical decision algorithm for ID selection based on curve characteristics and deformity stiffness is presented in Figure 5.

Comparison With Previous Systematic Reviews

Recent systematic reviews and meta-analyses have similarly concluded that higher ID does not consistently improve radiographic correction or patient-reported outcomes while increasing operative burden and implant-related costs [20,31,32]. The present narrative review complements these quantitative syntheses by incorporating recently published comparative studies and randomized trials and by providing a broader discussion of biomechanical evidence, economic considerations, variability in implant-density definitions, curve-specific decision-making, and practical implications for surgical planning. This broader perspective highlights areas where evidence remains inconsistent and identifies priorities for future research.

Clinical Implications and Future Directions

The findings of this review suggest that routine use of maximal pedicle screw ID in AIS surgery is not consistently supported by available data [7,8,13,15]. In many patients, effective 3D deformity correction can be achieved using lower-density constructs when screw placement is guided by curve magnitude, flexibility, Lenke classification, sagittal alignment, and correction objectives rather than by the use of uniform maximal fixation [9,10,13,20]. Emerging evidence increasingly supports the concept that thoughtful implant distribution and correction technique may be more important than absolute screw number alone [10,13].

From a practical standpoint, selective reduction in ID may improve operative efficiency, reduce blood loss, shorten operative duration, and substantially decrease implant-related expenditure [7,8,18,23]. These advantages are particularly relevant in the current era of economically sustainable spine care, where balancing surgical effectiveness with responsible resource utilization has become increasingly important. The economic implications may be even more significant in resource-constrained healthcare systems, where implant cost remains a major barrier to access for deformity surgery [7,18].

The available evidence also suggests that ID strategies should be individualized rather than universally applied [31-33]. Flexible curves, selective thoracic constructs, and less severe deformities may not require maximal instrumentation to achieve satisfactory correction, whereas larger or stiffer thoracic curves may occasionally benefit from increased fixation density in selected circumstances [13,18,19,21]. This reinforces the importance of tailoring instrumentation strategies to individual deformity characteristics and patient anatomy.

Despite the growing body of literature on ID, several important knowledge gaps remain. One major limitation in the literature is the lack of standardized definitions for low-density and high-density constructs, which limits meaningful comparison between investigations [10,13]. Future prospective studies using uniform ID definitions and standardized outcome reporting would improve comparability and strengthen the quality of evidence.

Additional research should also focus on patient-specific and curve-specific decision-making. Stratification by Lenke classification, curve flexibility, sagittal alignment, and correction technique may help clarify the clinical situations in which ID exerts meaningful influence on outcomes [13,19]. Long-term follow-up studies incorporating patient-reported outcomes, health-related quality of life measures, and maintenance of correction are similarly needed to better define the true clinical impact of ID beyond immediate radiographic correction [15,16].

Finally, further high-quality economic analyses are warranted. Future studies should incorporate not only implant costs, but also operative efficiency, blood loss, complication profiles, revision surgery, hospital utilization, and long-term healthcare expenditure [7,18,23]. Such data will be essential for developing evidence-based, patient-centered, and economically sustainable instrumentation strategies in AIS surgery [33,34]. A proposed clinical decision algorithm for ID selection based on curve characteristics and deformity stiffness is presented in Figure 5.

Limitations

This review is limited by the exclusion of non-English language studies, which may introduce language bias and under-represent research from regions such as Asia and South America. However, evidence suggests that language restrictions in reviews of conventional surgical interventions do not significantly alter conclusions. Additionally, heterogeneity in ID definitions, surgical techniques, and outcome reporting limits direct cross-study comparison. In addition, many included studies were retrospective observational investigations, limiting the ability to establish causal relationships between ID and clinical outcomes. Although recent systematic reviews and meta-analyses are available, the included studies remain heterogeneous with respect to implant-density definitions, curve characteristics, and outcome reporting, limiting direct comparison across studies.

Finally, as this was a narrative review, a formal methodological quality or risk-of-bias assessment of the included studies (e.g., Newcastle-Ottawa Scale, Risk Of Bias In Non-randomized Studies of Interventions (ROBINS-I), or A MeaSurement Tool to Assess systematic Reviews (AMSTAR)) was not performed. Consequently, the conclusions should be interpreted in the context of the predominantly observational nature of the available evidence. Future systematic reviews incorporating standardized quality appraisal and quantitative synthesis are warranted to strengthen the evidence base.

## Conclusions

The preponderance of evidence suggests that higher ID does not consistently confer superior radiographic or functional outcomes in AIS, while being associated with increased operative time, blood loss, and cost. Low-density pedicle screw constructs appear to offer a safe, effective, and economically favorable alternative in appropriately selected patients. Curve flexibility, Lenke classification, and surgical strategy exert greater influence on correction than screw density alone. A selective, curve-specific approach to ID appears more consistent with current literature than routine maximal pedicle screw instrumentation. Future prospective, standardized studies are required to define patient-specific ID strategies.
